# Noninvasive Imaging of *In Vivo* MuRF1 Expression during Muscle Atrophy

**DOI:** 10.1371/journal.pone.0094032

**Published:** 2014-04-07

**Authors:** Wei Li, Mark D. Claypool, Annabelle M. Friera, John McLaughlin, Kristen A. Baltgalvis, Ira J. Smith, Taisei Kinoshita, Kathy White, Wayne Lang, Guillermo Godinez, Donald G. Payan, Todd M. Kinsella

**Affiliations:** Discovery Research, Rigel Pharmaceuticals Inc., South San Francisco, California, United States of America; Leibniz Institute for Age Research - Fritz Lipmann Institute (FLI), Germany

## Abstract

Numerous human diseases can lead to atrophy of skeletal muscle, and loss of this tissue has been correlated with increased mortality and morbidity rates. Clinically addressing muscle atrophy remains an unmet medical need, and the development of preclinical tools to assist drug discovery and basic research in this effort is important for advancing this goal. In this report, we describe the development of a bioluminescent gene reporter rat, based on the zinc finger nuclease-targeted insertion of a bicistronic luciferase reporter into the 3′ untranslated region of a muscle specific E3 ubiquitin ligase gene, MuRF1 (Trim63). In longitudinal studies, we noninvasively assess atrophy-related expression of this reporter in three distinct models of muscle loss (sciatic denervation, hindlimb unloading and dexamethasone-treatment) and show that these animals are capable of generating refined detail on *in vivo* MuRF1 expression with high temporal and anatomical resolution.

## Introduction

MuRF1 ubiquitin ligase was discovered more than a decade ago as one of two muscle specific ubiquitin ligases selectively induced during skeletal muscle atrophy [1]. Since then, numerous laboratories have investigated MuRF1 expression and found it to be upregulated, both at the mRNA and protein level, during a wide range of pathological conditions associated with muscle atrophy [2]–[5]. MuRF1 knockout and dominant-negative knockin mice display significant resistance to muscle atrophy in a variety of model systems, suggesting MuRF1 plays a mechanistically important role in initiating and/or sustaining atrophy [1], [6]–[9]. The central role of MuRF1 during skeletal muscle atrophy and its near universal upregulation in diseases associated with muscle loss, suggest it is an excellent biomarker for skeletal muscle atrophy.

Standard methods for monitoring gene expression, such as northern blot analysis, quantitative RT-PCR (qRT-PCR) or western blotting are limited in many regards by the fact that these techniques require procedures that are terminal, labor intensive, require large numbers of animals for serial measurements and are generally incompatible with longitudinal studies. Previously, two other transgenic models with MuRF1 expression-related readouts have been described and each has provided important foundational insights into the role and/or regulation of MuRF1 during skeletal muscle atrophy [1], [10]. However, the reporter capabilities of both of these model systems was limited in important ways related to how each was configured, including the use of beta-galactosidase as a reporter, which requires sacrificing animals in order to stain tissues for the presence of the transgene. The first of these model systems was primarily constructed for the purposes of studying the impact of knocking out the MuRF1 gene in mice, but these animals none-the-less had reporter functionality since a *LacZ/neomycin* cassette was utilized to replace the first 5 exons of MuRF1 [1]. However, from a reporter standpoint, the intentional abrogation of MuRF1 expression in this configuration does confound integrated functional studies since it deprives muscles of a critical atrophy effector protein, thereby rendering animals significantly resistant to muscle atrophy. In the second approach, a beta-galactosidase expression cassette was assembled using several hundred base pairs of the MuRF1 promoter fused to the hsp68 minimal promoter, and randomly integrated into the genome of mice [10]. Although this transgene was able to drive reporter expression in some myofibers, it was unclear if the full regulatory scheme utilized to control selective MuRF1 gene expression in muscle was entirely localized to the promoter fragment utilized, and this reporter system was not characterized as to how closely it reflected endogenous MuRF1 induction. Furthermore, beta-galactosidase detection methods require sacrifice of animals for reporter readout, limiting the utility of this system as described as above.

Whole animal imaging of bioluminescent reporters is a sensitive, quantitative and powerful tool for noninvasively following biological processes *in vivo* [11], [12]. A host of preclinical animal models have taken advantage of this imaging modality to generate important insights into disease and basic biological mechanisms [13], [14]. However, this technology has not yet been successfully applied to a transgenic animal model that is universally reflective of skeletal muscle atrophy. We describe here, the use of zinc finger nuclease technology (ZFN) [15], [16], to knockin a luciferase reporter directly downstream of MuRF1 coding sequences in rats, leaving endogenous MuRF1 gene expression intact and bicistronically linking it to the inserted reporter through a hepatitis C IRES (HCV-IRES). The resulting knockin rat line, MuRF1-hiLUCs, has reporter characteristics that are fully reflective of endogenous MuRF1 gene expression, can be used as a universal biomarker of skeletal muscle atrophy *in vivo*, and has a bioluminescent readout compatible with whole animal imaging modalities. New details about the temporal and spatial pattern of MuRF1 induction during denervation-, hindlimb unloading- and dexamethasone-mediated muscle atrophy are presented. Our results validate the MuRF1-hiLUCs rat as a noninvasive, quantitative, and versatile biomarker system that can be applied to both basic research and the development of therapeutics for skeletal muscle atrophy. To the best of our knowledge, this is the first reported use of ZFN technology to create a knockin rat with reporter capabilities.

## Methods

### Ethics Statement

Animal studies were conducted in strict accordance with the NIH guidelines for humane treatment of animals and approved by the IACUC at Rigel Pharmaceuticals Inc.

### Generation of the MuRF1-hiLUCs reporter rat

The MuRF1-hiLUCs reporter rat was generated by inserting a donor cassette consisting of a HCV IRES-Luciferase sequence into the last intron of the MuRF1 gene using the ZFN technology. The donor cassette also contains (5′ to the HCV_IRES sequence) a reconstructed last exon of the MuRF1 gene, including the branch point and splicing acceptor site, to keep the endogenous MuRF1 gene structure intact. The ZFN target site sequence is: cgccacagctcaaaaggccctgggtcctctcca. The validated ZFNs and the donor plasmid were delivered via microinjection into the nucleus of a fertilized single cell rat embryo. The presence of targeted insertion and absence of random integration in the founder rats were identified using PCR analysis, and later confirmed by genomic sequencing of the donor cassette and the junctions. The PCR primers used were:

p1: GCTCAGCAAGGAGGTAGGTG, p2: TATCTAGCCCCCTGCCTTTT

p3: GAGGCTGCACGACACTCATA, p7: GAGCCAGCAGATTTCAAAGG

p4: CCTGCTGGCACTACAGATGC, p8: AGCCAGCAGATAAGCTCTCG

p5: AAGGGCGAAAAACCGTCTAT, p6: GCTGAAGCCAGTTACCTTCG

Three knockin founder lines were established on the genetic background of Sprague-Dawley rats and line 30 was selected for expansion. All rats used for this manuscript were heterozygous progenies derived from line 30 unless specified otherwise. Design, validation and assembly of the ZFN pairs; microinjection and screening of the F0 generation; establishment and subsequent genotyping and expansion of the founder lines were all performed at the Sigma Advanced Genetic Engineering (SAGE) Labs as a custom service.

### *In vivo* and *ex vivo* bioluminescence imaging

All *in vivo* imaging was performed using the IVIS-Spectrum system (Caliper Life Sciences). Rats were given 350 μl to 700 μl of Luciferin Ultra™ (depending on body weights) via intraperitoneal (i.p.) injection, for at least 6 minutes before anesthesia with 2.5% isoflurane and 100% oxygen at a flow rate of 1.5 L/min. Rats were first imaged for 3 seconds under the Fluorescent filter (Ex = 745, Em = 800) to confirm successful luciferin injection. Rats were then imaged for 3 to 5 mins under luminescent settings for maximal sensitivity (f/stop1) with medium binning. Exposure times were kept constant within each study. For all image data used in the figures, the luminescent intensities were expressed as radiance or photons/second/ centimeter squared/steradian (p/s/cm2/sr). For *ex vivo* imaging of tissues, rats were injected with Luciferin via i.p. before the tissues were dissected out and placed on a 6-well tissue culture dish for imaging in the IVIS Spectrum. Imaging settings were the same as for *in vivo* imaging.

### Image analysis

All image data were analyzed using the Living Image software 4.3.1 (Caliper Life Sciences). All luminescent intensities were normalized to the fluorescent intensity of the Luciferin injection in order to correct for the delivery efficiency of the luciferase substrate. ROIs were first defined using the baseline images and replicated for the same rat throughout different time courses of a study. For any given analysis, all images were adjusted to the same scale of minimum and maximum luminescent intensity. Fold induction of luminescent intensities were calculated over the baseline values.

### Skeletal muscle atrophy models

For glucocorticoid-induced MuRF1-hiLUCs reporter activity, rats were administered 600 ug/kg body weight of dexamethasone or saline via i.p. injection for three consecutive days and left to recover. For muscle weight, CSA and gene expression analyses, rats were given 1.5 mg/kg body weight of dexamethasone daily in drinking water for 7 days.

Rat hindlimb denervation was performed as described by Csukly et al. 2006 [17], with slight modifications. A 2 cm skin incision was made in the mid-posterolateral area of the thigh. A 10–12 mm pocket was made along the separation of the muscle groups or bundles and deepened by blunt dissection to expose the sciatic nerve. A 5–10 mm segment of the sciatic nerve was then removed using very fine scissors (micro-iris, Vannas). For the accompanying sham operation, the sciatic nerve was exposed but not transected. The muscle groups were approximated and returned to close up the created pocket, and the skin incision was closed with interrupted stitches using absorbable sutures. The left hindlimb was denervated and the right hindlimb served as a non-denervated internal control.

Female rats 7–8 weeks of age with body weights ranging from 200–250 grams were used in the HLU study. Rat HLU was carried out by methods described by Arbogast et al. 2007 [18], with slight modifications. The tail was cleaned with an alcohol prep pad and air-dried. Compound Tincture of Benzoin was applied before an elastic adhesive bandage (Tensoplast) was wrapped circumferentially around the proximal 2/3 of the tail. A metal ring was attached by adhesive bandage, and then fastened to a steel rod mounted across the top of the cage via rotatable fishing connecters. The system allows the animals to move freely about the cage while preventing the hindlimbs from touching the floor or walls. Control rats had bandages around their tails but were loaded, and singly housed in the same cage environment as the unloaded rats.

### Histology and CSA analysis

Muscles were removed while rats were under anesthesia with 3% isoflurane and were immediately frozen in liquid nitrogen chilled isopentane and later embedded in OCT. Frozen muscles were cut with a cryostat to generate 6-um-thick cross sections. H&E staining and immunofluorescent staining with an anti-laminin antibody (Sigma L9393, 1∶200 dilution) were performed using the cryosections. Slides with laminin staining were digitally imaged with a 4X objective to capture each entire tissue section using a Molecular Devices IXMicro fluorescent microscope. For both gastrocnemius and soleus muscles 6 sections per animal were analyzed. Gastrocnemius averaged 15 images per section and Soleus 3 images per section using a CMOS camera with a 2160×2160 pixel field of view. Image analysis was performed with CellProfiler. Images were analyzed separately, not as a single montage of the entire section. Thresholding was performed using Otsu's method minimizing weighted variance with three classes. The middle class was assigned to background. Morphological techniques were applied to provide outlines for each fiber. Images with poor or incomplete thresholding were discarded from the analysis by manual inspection. Tissue sections usually required multiple image fields for complete coverage and all image results for each section were summed and then results for each section were averaged across sections for each animal. Muscle fiber size is reported in um^2^.

### Taqman gene expression assay and Panomics QuantiGene multiplex assay for RNA expression

Total RNA was extracted from the gastrocnemius muscle using Trizol Plus reagent (Life technologies). Quantitative RT-PCR was performed using custom Taqman gene expression assays (Life technologies). The primer sequences used were the following:

Forward primer for MuRF1-hiLUCs: GAGGGCGTGTCCACAGA

Reverse primer for MuRF1-hiLUCs: AAGCTGGTCCAGGATTTGTAGTG

Forward primer for Luciferase: CGCCGAGTACTTCGAGATGA

Reverse primer for Luciferase: GCCCATAGCGCTTCATAGCT

MuRF1 mRNA was amplified using the Taqman gene expression assay Rn_00590197.

20–30 mg of gastrocnemius muscles were homogenized and the lysates were used in a custom designed Panomics QuantiGene multiplex assay. Each multiplex contains probes for 4 target RNAs including: MuRF1, Atrogin-1, HDAC4 and Myogenin. Also included in the same multiplex were 3 housekeeping genes (Hprt1, Gusb and Hmbs) for data normalization.

### Antibodies

Antibody against MuRF1 was purchased from ECM biosciences (MP3401) and pan-actin antibody from cell signaling (#12748).

### Statistics

Statistical analyses were performed with Graphpad Prism 6.0, using unpaired, two-tailed t test, ordinary one-way ANOVA with Tukey's or Sidak's multiple comparisons test or ordinary two-way ANOVA with Tukey's or Dunnett's multiple comparisons test. Normal distribution of data was confirmed using either the D'Agostino & Pearson omnibus normality test (N>8) or the Shapiro-Wilk normality test (N>7). *P<0.05 was considered as statistically significant. Data represent means + SEM.

## Results

### Generation of the MuRF1-hiLUCs reporter rat

**Figure 1:**
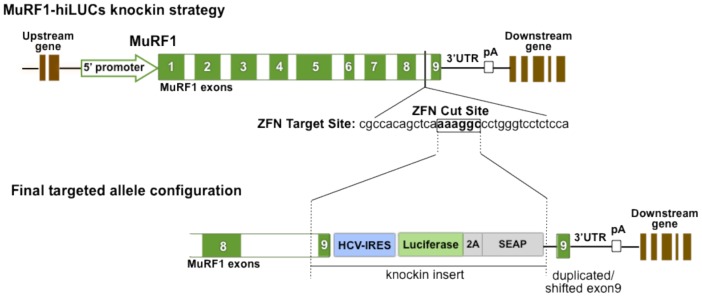
Targeted insertion strategy for creating the MuRF1-hiLUCs knockin rat. An HCV-IRES-Luciferase-2A-SEAP reporter was inserted into the last intron of the endogenous MuRF1 gene via ZFN targeting (exons green with white numbers). In order to preserve the MuRF1 endogenous coding sequence and properly align the reporter ORF, a reconstructed last exon (exon 9) of the MuRF1 gene was included in the insertion cassette. This resulted in the shifting down of the original genomic exon 9 after knockin of the reporter cassette while leaving the 3′UTR intact. Founder #30 and two other founders were identified as valid MuRF1-hiLUCs knockin founder lines.

Previous attempts by us to generate MuRF1 promoter constructs and engineer BAC reporter clones, in preparation for the creation of a transgenic MuRF1 reporter system, suggested that this gene was particularly sensitive to manipulations near its 5′ region and that the full genomic regulatory scheme controlling its expression was complex, and not necessarily entirely encapsulated in 5′ promoter fragments of up to 4 kb in length (data not shown). We therefore turned to ZFN-based methodologies to knockin an HCV-IRES-Luciferase-2A-SEAP reporter cassette immediately downstream of the endogenous MuRF1 coding sequences in rats. This configuration allowed bicistronic expression of the MuRF1 gene and the reporter from the same transcript, both under the control of the endogenous MuRF1 regulatory apparatus in its native genomic context (Figure 1). Luciferase was chosen as the primary reporter in order to facilitate bioluminescent imaging and this was further linked, through a foot-and-mouth disease virus 2A ribosomal skipping element (FMDV 2A), to secreted alkaline phosphatase (SEAP) in order to introduce a secondary, blood-based reporter functionality. In contrast to the primary luciferase reporter described throughout this manuscript, SEAP activity was consistently below the limit of detection (data not shown). This finding was in line with some other literature reports that encountered challenges when using this enzyme for *in vivo* readouts [13] and, therefore, additional SEAP data is not further presented. The targeted insertion of the reporter cassette and the intactness of the endogenous MuRF1 coding sequences were verified by PCR, genomic sequencing and western blot analyses (Figure S1 and S2). Correct splicing of the MuRF1-hiLUCs allele was verified by allele specific qRT-PCR (Figure S3). In this analysis, the MuRF1-hiLUCs mRNA showed the same level of basal and induced expression (with dexamethasone treatment) as the luciferase mRNA, indicating that all luciferase-containing message was derived from the MuRF1-hiLUCs transcript, driven by the endogenous MuRF1 promoter. Heterozygous and homozygous reporter rats were born in Mendelian ratios and phenotypically indistinguishable from their *wild-type* littermates.

### Characterization of the MuRF1-hiLUCs reporter rat in a model of glucocorticoid-induced muscle atrophy

**Figure 2:**
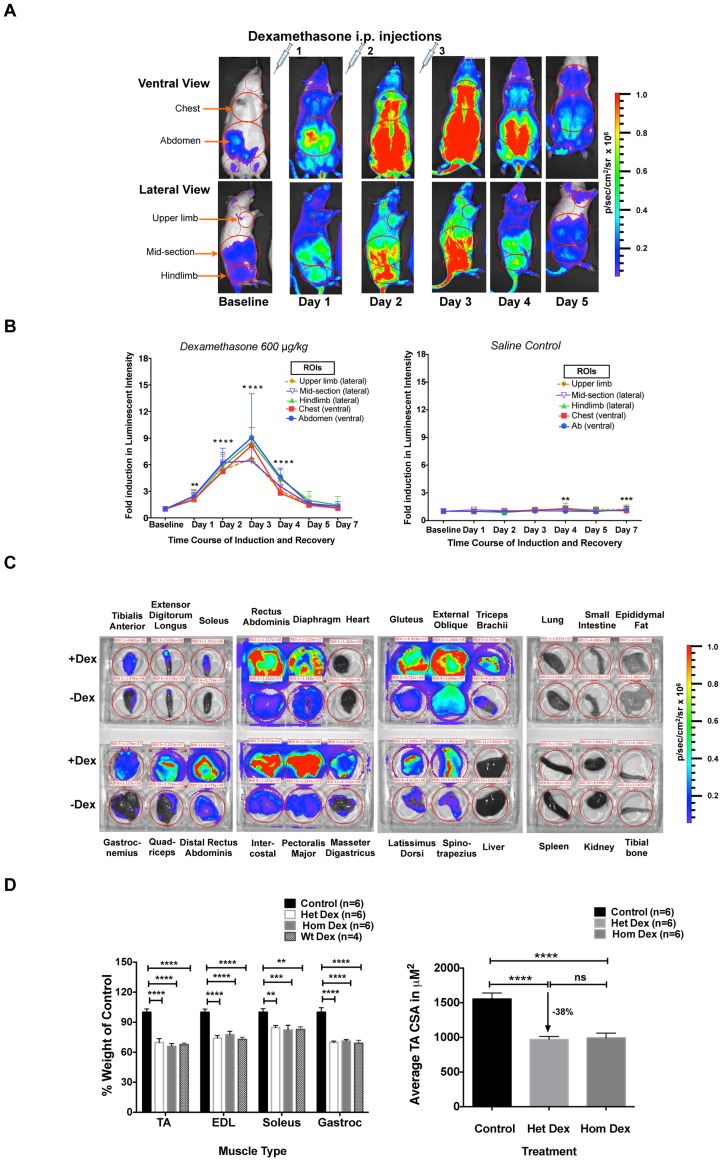
Time course of the MuRF1-hiLUCs reporter induction and extent of muscle atrophy following dexamethasone treatment. (**A**) MuRF1-hiLUCs reporter rats were given either 600 ug/kg of dexamethasone or saline via i.p. for three consecutives days. *In vivo* images of ventral and right lateral views were acquired before treatments (baseline) and then daily thereafter, starting 24 hours after the first dose, until day 7. Shown are the ventral and lateral images of a representative rat from day 1- day 5. (**B**) Quantitation of the fold induction of luminescent intensity of the MuRF1-hiLUCs reporter during the time course of the dexamethasone (n = 10 for day 1–5, n = 9 for day 7) or saline (n = 8) treatment. Five distinct ROIs were chosen for the quantitation. Fold inductions were calculated over baseline values. Data are presented as means (symbols) + SEM (lines). Statistics were performed using ordinary two-way ANOVA with Dunnett's multiple comparisons test. *P<0.05, **P<0.01, ***P<0.001, ****P<0.0001. (**C**) Induction of the MuRF1-hiLUCs reporter in dissected skeletal muscles after dexamethasone treatment. MuRF1-hiLUCs reporter rats treated identically as indicated above but then sacrificed on day 3, one day after the last dosing. Skeletal muscles and otherwise indicated tissues were dissected out immediately after the luciferin injection and placed in a 6-well dish for visualization. The experiment shown is representative of three independent experiments. (**D**) Quantitation of the extent of hindlimb muscle atrophy in MuRF1-hiLUCs reporter rat after 7 days of dexamethasone treatment. All muscle weights were normalized to body weights and presented as percent of normalized muscle weights from control rats. CSA analysis for the tibialis anterior muscle was performed as described in the methods. Data are presented as mean (symbols) + SEM (lines). Statistics were performed using one-way or 2way ANOVA with Tukey's multiple comparisons test. **P<0.01, ***P<0.001, ****P<0.0001.

Synthetic glucocorticoids, such as dexamethasone, are powerful inducers of both MuRF1 expression and skeletal muscle atrophy [1], and participate in the direct induction of MuRF1 through multiple glucocorticoid receptor binding sites located within the MuRF1 promoter [19], [20] Additionally, MuRF1 ubiquitin ligase activity has been linked to degradation of myosin heavy chain upon dexamethasone treatment, and muscle atrophy is significantly attenuated in MuRF1 knockout mice subjected to dexamethasone [7], [9]. Collectively, this data prompted us to characterize the MuRF1-hiLUCs reporter line during dexamethasone treatment. F1 heterozygous MuRF1-hiLUCs reporter rats were given once daily doses of 600 ug/kg body weight dexamethasone via intraperitoneal (i.p.) injection for three consecutive days, or were given saline at the same volume/body weight ratio as a control. Baseline reporter expression was measured prior to treatment and then at 24 hour intervals after the first dose for a total of 7 days, with the exception that no image was taken on day 6. Dexamethasone treatment led to global induction of the MuRF1-hiLUCs reporter in areas anatomically consistent with skeletal muscle. The luciferase activity increased with repeated dosing, peaking on day 3, which was one day after the last dose and diminishing thereafter with kinetics similar to the initial rise (Figure 2, A and B, Figure S4, A and B). This induction was specific to dexamethasone treatment since only background levels of luciferase activity were detected in reporter rats injected with saline (Figure 2B). Interestingly, induction was most prominent at the site of the dexamethasone i.p. injection (mid abdominal area) and persisted in this location the longest after the cessation of dosing (Figure 2A). Induction of the endogenous MuRF1 as well as the luciferase mRNA by dexamethasone treatment was further confirmed using qRT-PCR (Figure S4C). In order to confirm the tissue specificity of MuRF1-hiLUCs induction, we dissected various tissues from dexamethasone vs. saline treated reporter rats and imaged them *ex vivo* 3 days after treatment. MuRF1-hiLUCs induction was highly restricted to skeletal muscles relative to other tissues, but broadly upregulated across different muscle types (Figure 2C). Neither baseline nor dexamethasone-induced expression was apparent in cardiac muscle (heart), smooth muscle (small intestine), fat, bone or other organs (Figure 2C).

Both homozygous and heterozygous MuRF1-hiLUCs reporter rats underwent atrophy at a similar level as that of their *wild-type* littermates following dexamethasone treatment (Figure 2D), indicating that insertion of the reporter into the 3′ UTR of the MuRF1 gene did not functionally compromise the ability of these animals to undergo atrophy.

### Characterization of the MuRF1-hiLUCs reporter rats during hindlimb denervation

**Figure 3:**
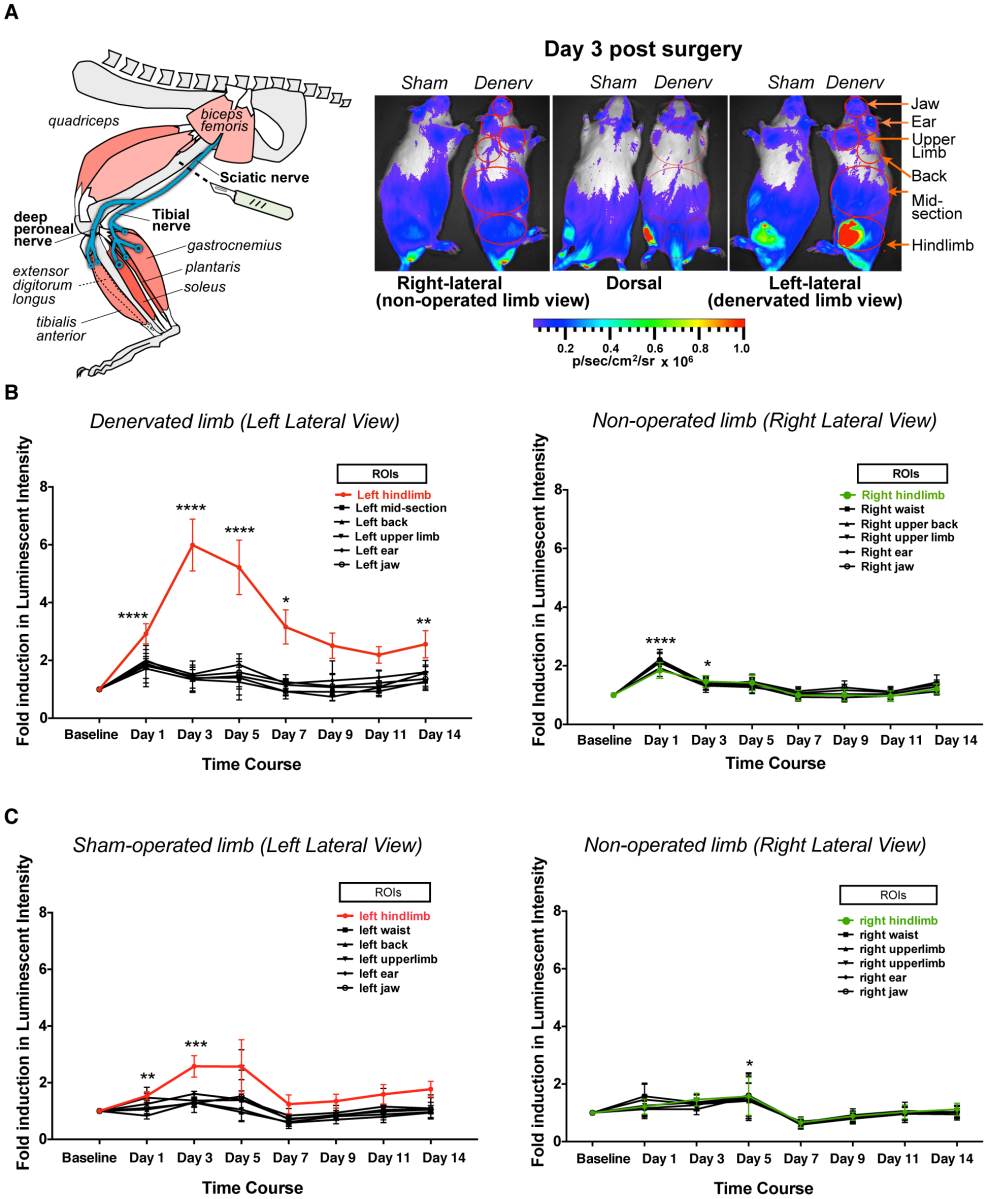
Time course of the MuRF1-hiLUCs reporter induction during a 14-day hindlimb denervation study. (**A**) Left hindlimbs of the MuRF1-hiLUCs reporter rats underwent sciatic nerve denervation or sham surgeries (schematic). *In vivo* images of the ventral, left lateral, right lateral and dorsal views were acquired before the surgeries (baseline) and every other day thereafter from day 1 to day 14. Shown are the dorsal and lateral images of a denervated rat and a sham-operated rat on day 3 post-surgery. (**B**) Quantitation of the fold induction of luminescent intensity of the MuRF1-hiLUCs reporter during the time course of the denervation (n = 8). Six distinct ROIs from either the denervated side (left lateral view) or the control side (right lateral view) were chosen for the quantitation. Fold inductions were calculated over baseline values. Data are presented as means (symbols) + SEM (lines). Statistics were performed using ordinary two-way ANOVA with Dunnett's multiple comparisons test. *P<0.05, **P<0.01, ***P<0.001, ****P<0.0001. (**C**) Quantitation of the fold induction of luminescent intensity of the MuRF1-hiLUCs reporter during the time course of the sham operation (n = 4). Data were quantified the same way as from the denervated rats.

**Figure 4:**
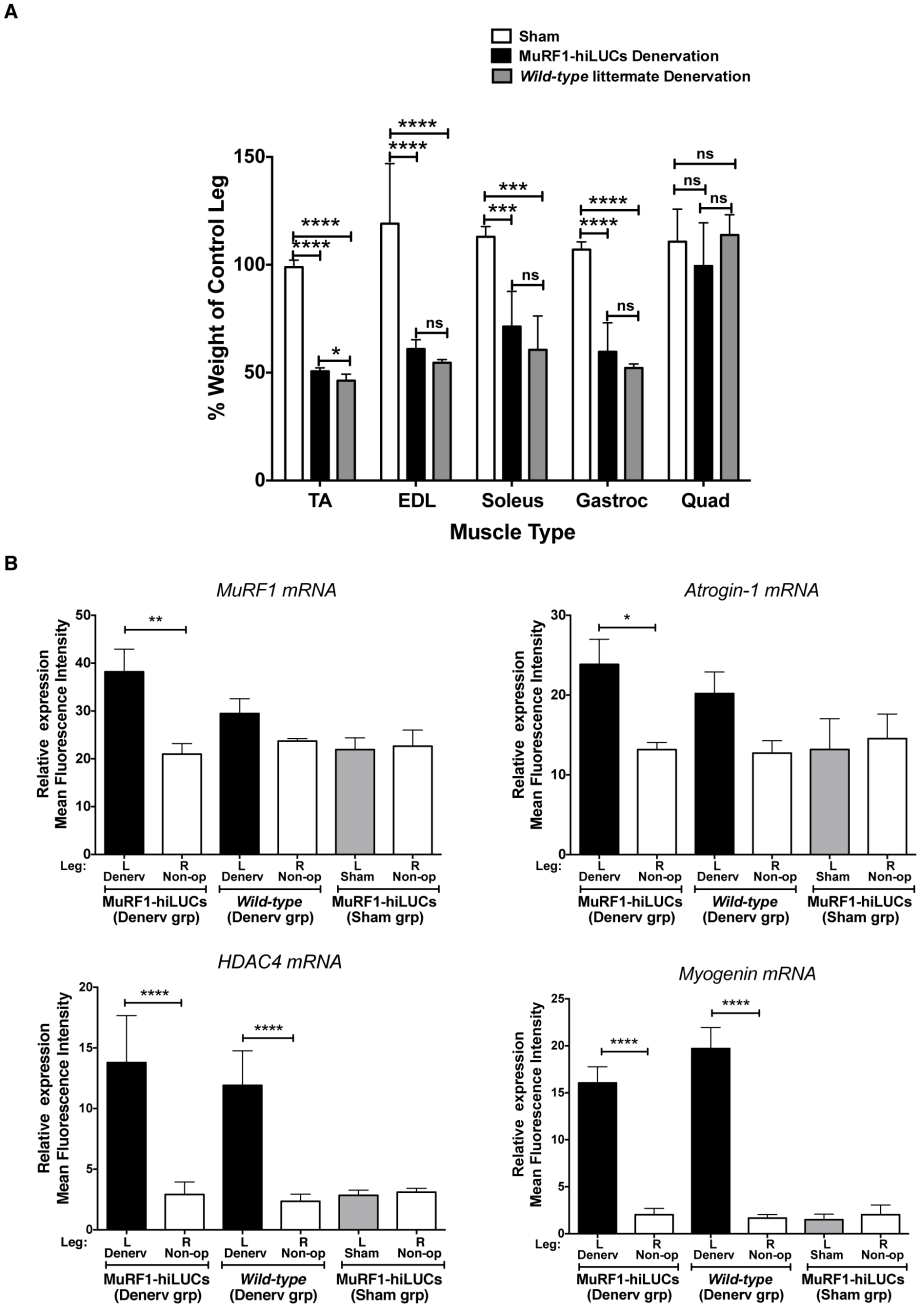
Atrophic response to denervation in MuRF1-hiLUCs reporter rats vs. the *wild-type* littermates. (**A**) Extent of muscle atrophy in denervated vs. sham-operated rats after 14 days of denervation. Hindlimb muscles were harvested on day 14 after surgeries from denervated (n = 8) or sham operated (n = 4) MuRF1-hiLUCs reporter rats and their *wild-type* littermates that underwent denervation (n = 8). Muscle weights from the denervated leg were normalized to the muscle weights from the contralateral leg of the same rat, since no compensatory hypertrophy was detected in the contralateral leg (Figure S6). Data are presented as means (symbols) + SEM (lines). Statistics were performed using ordinary one-way ANOVA with Tukey's multiple comparisons test. *P<0.05, ***P<0.001, ****P<0.0001. (**B**) Endogenous MuRF1 mRNA induction in the MURF1-hiLUCs reporter rats compared to *wild-type* littermates. Muscles from both hindlimbs of the sham-operated and denervated MuRF1-hiLUCs reporter rats and their denervated *wild-type* littermates were harvested on day 14 after the surgeries. 20∼30 mg of the gastrocnemius muscles were homogenized and subjected to the Panomics Quantigene multiplex assay. Results were normalized to the geometric mean of three house keeping genes: hprt1, gusb and hmbs. Statistics were performed using one-way ANOVA with Tukey's multiple comparisons test. *P<0.05, **P<0.01, ****P<0.0001.

Denervation is perhaps one of the most robust inducers of skeletal muscle atrophy known, primarily due to the complete cessation of contractile activity. MuRF1 ubiquitin ligase is strongly induced during denervation and its activity is essential for the atrophic response [1], [6], [21]. For example, MuRF1 knockout mice underwent 36% less atrophy after 14 days of denervation, and degradation of the thick filament components in myofibrils was impaired in MuRF1 dominant negative knockin mice [1], [6]. To further validate the MuRF1-hiLUCs reporter line, we utilized a 14-day, unilateral, sciatic nerve transection model to selectively denervate the gastrocnemius, soleus, tibialis anterior and extensor digitalis longus muscles of the hindlimb, without affecting quadriceps muscles of the same leg since these muscles are not innervated by the sciatic nerve (Figure 3A schematic). As demonstrated in Figure 3, denervation of the left hindlimb resulted in robust induction of the MuRF1-hiLUCs reporter relative to sham-operated controls or the non-operated contralateral leg from the same animal. A significantly lower, but noticeable level of MuRF1-hiLUCs expression could be attributed to localized trauma from the surgery itself, since sham operated controls subjected to identical incision and blunt dissection methods displayed some reporter signal for the first three days post-procedure (Figure 3, A and C). In denervated muscle, induction of the reporter over baseline occurred rapidly and was elevated by 3 fold within 24 hours of surgery. Peak levels of reporter expression occurred at day 3 post-denervation and then steadily declined until day 7, leveling off at 2–3 fold over baseline for the remainder of the 14 day observation period (Figure 3, B and C, Figure S5). Dissected muscle weights from day 14 post-denervation demonstrated that MuRF1-hiLUCs rats underwent muscle atrophy at levels that were comparable to *wild-type* littermate controls (Figure 4A). Likewise, qRT-PCR analysis of endogenous MuRF1 gene expression at day 14, a point at which reporter signal in the denervated leg had declined from its peak to approximately 2 fold above the non-operated leg, demonstrated that MuRF1-hiLUCs rats were not diminished in their ability to induce MuRF1 relative to *wild-type* littermates (Figure 4B). Other genes known to be highly induced by denervation [1], [10], [22], [23] were similarly increased in MuRF1-hiLUCs rats and *wild-type* littermate controls at day 14 in these same muscles (Figure 4B), further supporting the conclusion that the MuRF1-hiLUCs reporter rats had fully functional atrophic responses.

### Characterization of the MuRF1-hiLUCs reporter rat during hindlimb unloading

**Figure 5:**
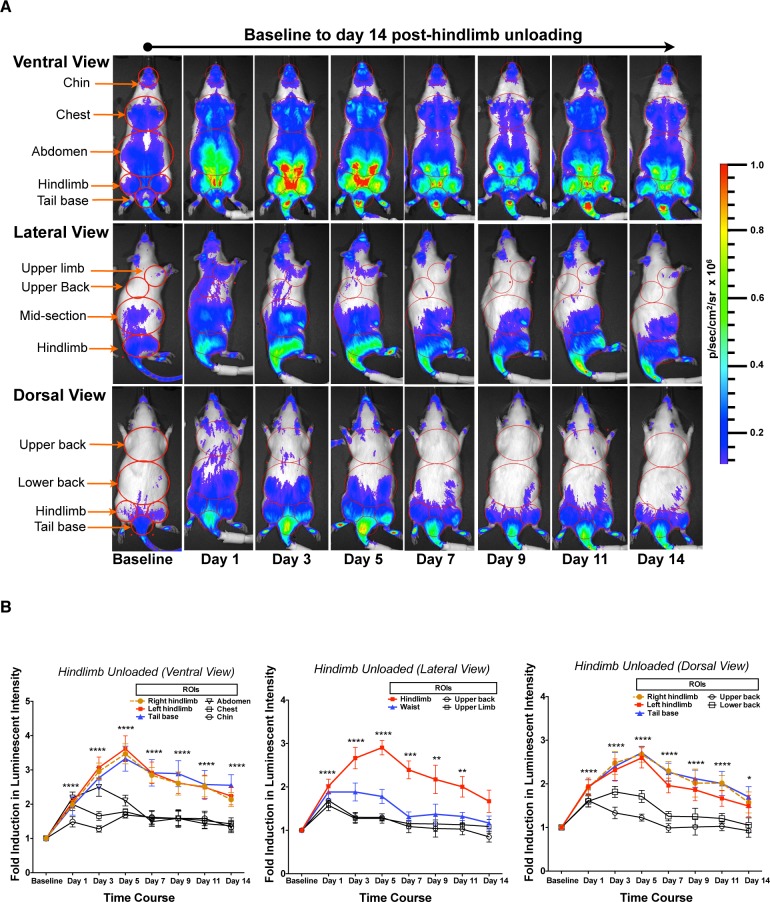
Time course of the MuRF1-hiLUCs reporter induction during a 14-day HLU study. MuRF1-hiLUCs reporter rats (n = 8) were hindlimb unloaded (HLU) for 14 days. *In vivo* images of the ventral, right lateral and dorsal views of the rats were acquired before the unloading (baseline) and every other day thereafter from day 1 to day 14. (**A**) Ventral, lateral and dorsal images of a representative HLU rat throughout the time course of the unloading. (**B**) ROIs from all three views were used to quantify the fold induction of the MuRF1-hiLUCs reporter during the time course of the unloading (n = 8). Fold inductions of the luminescent intensities were calculated over baseline values. Data are presented as means (symbols) + SEM (lines). Statistics were performed using ordinary two-way ANOVA with Dunnett's multiple comparisons test. *P<0.05, **P<0.01, ***P<0.001, ****P<0.0001.

**Figure 6:**
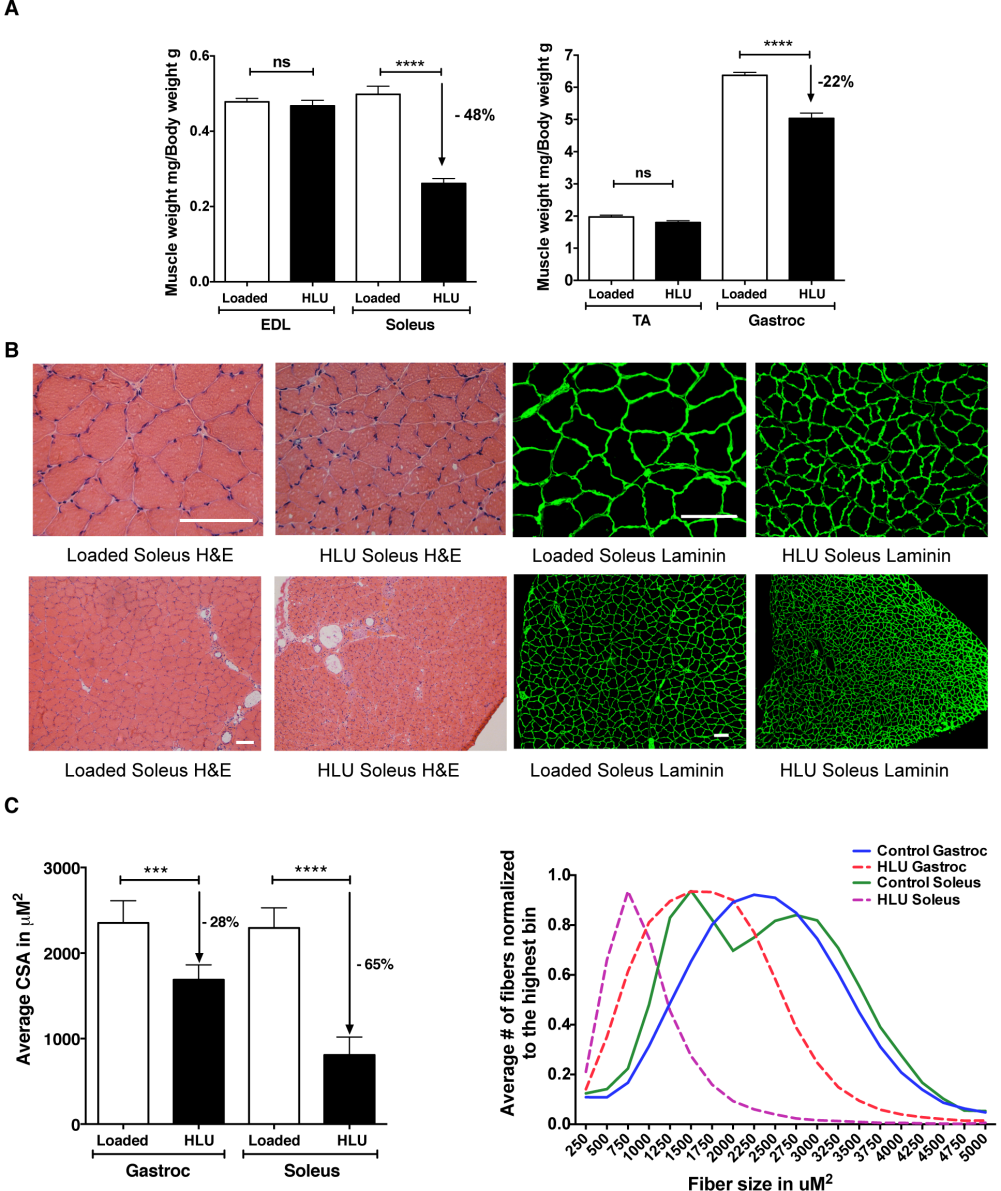
Extent of muscle atrophy in hindlimb unloaded vs. control rats after 14 day of HLU. (**A**) Hindlimb muscles were harvested on day 14 from unloaded (n = 8) or loaded control (n = 6) MuRF1-hiLUCs reporter rats. Muscle weights were normalized to body weights. Data are presented as means (symbols) + SEM (lines). Statistics were performed using unpaired, two-tailed t test. ****P<0.0001. (**B**) Histological analyses of the hindlimb muscles from 14-day HLU rats vs. loaded control rats. Both H&E and laminin staining showed dramatic reductions in the CSA of the HLU vs. the control soleus muscles. Scale bars: 100 uM. (**C**) Comparison of the average CSA and fiber size distribution of the HLU vs. the loaded control soleus and gastrocnemius muscles. CSA analysis was performed using an internally developed algorithm as described in the methods. An average of 57,000 fibers (6 cross-sections) were analyzed for each gastrocnemius muscle and 3600 fibers (6 cross-sections) were analyzed for each soleus muscle. Statistics were performed using ordinary one-way ANOVA with Sidak's multiple comparisons test. ***P<0.01, ****P<0.0001.

Hindlimb unloading (HLU) is a “reduced muscle use” model of atrophy in which the rear quarter of an animal is suspended above the ground for extended periods of time in order to prevent ambulation related-muscle contractions of the hindlimbs. This commonly used model was originally conceived to simulate skeletal muscle atrophy associated with the weightlessness of spaceflight, but is now more often utilized as a general model of muscle disuse atrophy [24]–[26]. Since skeletal muscle atrophy in this model system tends to be more gradual and less pronounced than denervation-based responses [1], we characterized the MuRF1-hiLUCs reporter rat during 14 days of HLU. As demonstrated in Figure 5, peak reporter expression was both lower and delayed relative to that observed during denervation experiments (3 fold peak at day 5 in HLU vs. 6.5 fold peak on day 3 in denervation). Anatomically, reporter induction appeared most concentrated in the tail, hindlimb and lower abdominal muscles. MuRF1 reporter induction in the tail and lower abdominal muscles during HLU has not been previously described in the literature, highlighting the spatial and temporal refinements possible with this reporter system. Interestingly, a milder, more transient but noticeable induction of reporter was also present in the musculature of the upper body, a previously unappreciated detail possibly related to the initial stress of the procedure (Figure 5, A and B, Table S1). After peak induction at day 5, MuRF1-hiLUCs expression slowly declined for the remainder of the 14-day experiment (Figure 5B and Table S1) but never dropped below baseline levels. No induction of MuRF1-hiLUCs was observed in the loaded control rats during the whole course of the study (Figure S7). After 14 days of HLU, and in line with expectations based on previously published reports [27], [28], gastrocnemius and soleus muscles underwent severe atrophy compared to control rats (22% and 48%, respectively), whereas atrophy in the tibialis anterior (TA) and extensor digitorum longus (EDL) muscles was insignificant (Figure 6A). Both H&E and laminin staining revealed a decrease in the cross sectional area (CSA) in the unloaded rats compared to loaded controls, confirming that the loss in muscle mass was due to atrophy of the muscle fibers (Figure 6, B and C).

### Comparison of the induction patterns of MuRF1-hiLUCs reporter in three different muscle atrophy models

**Figure 7:**
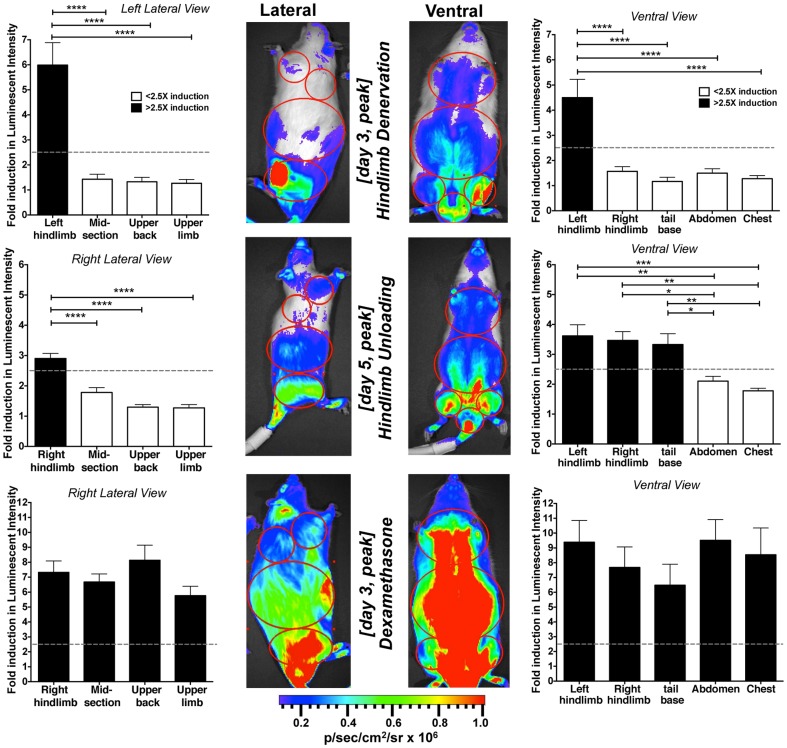
Comparison of peak MuRF1-hiLUCs induction in different anatomical regions in response to different atrophic stimuli. Levels of MuRF1-hiLUCs reporter induction were quantified in four or five muscle groups, depending on the view, from all three of the atrophy models investigated, throughout the time course of the studies (n = 8 for denervation and HLU, n = 10 for dexamethasone treatment). Graph bars correspond to day of peak reporter induction. Dotted lines represent 2.5 fold induction in luminescence. Differences in the level of MuRF1-hiLUCs reporter induction between muscle groups were analyzed using ordinary one-way ANOVA with Tukey's multiple comparisons test. *P<0.05, **P<0.01, ***P<0.001, ****P<0.0001. Data are presented as means (symbols) + SEM (lines).

Since each of the three atrophy models examined demonstrated differences in the temporal pattern, anatomical location and strength of reporter induction, we compiled a side-by-side comparison of MuRF1-hiLUCs expression for each of the main muscle groups affected over the course of the three studies (Figure 7). Dexamethasone treatment caused the most intense and broadly distributed expression pattern of the reporter relative to the other model systems. Hindlimb denervation had the second most robust induction pattern and was clearly the most selective. HLU had the slowest time until peak expression with the lowest level of reporter induction and was not restricted exclusively to the hindlimbs since strong induction was also observed in the tail and lower abdominal muscles (see also Figure 5, A and B).

## Discussion

It is widely recognized that excessive loss of skeletal muscle can significantly decrease survival rates in the context of chronic disease and it can also contribute to functional decline in settings where strength and endurance fall below basic daily needs [26], [29]–[31]. There is a compelling need to develop therapeutics to clinically address skeletal muscle atrophy, and preclinical animal models are important tools for advancing this goal. Prior to the creation of the MuRF1-hiLUCs reporter rat described here, noninvasively tracking *in vivo* induction and/or sustainment of atrophy-associated MuRF1 expression by bioluminescent imaging was not possible. The noninvasive nature of this technology, coupled with sensitive and quantitative detection, offers numerous advantages over other approaches and allowed detailed kinetic analysis of atrophy-associated MuRF1 induction with less labor, fewer animals and with less variability, since each animal could be compared to its own baseline. The overall kinetics of MuRF1-hiLUCs induction observed in each of the atrophy models examined was in accordance with previously published data generated on endogenous MuRF1 expression using northern blot analysis and qRT-PCR [1], [3], attesting to the ability of the reporter to accurately reflect atrophy-associated MuRF1 expression. Furthermore, these reporter rats were capable of providing a comprehensive picture of the atrophic process, with high temporal and anatomical resolution, without limiting the analysis to only those muscles that are procedurally easiest to dissect, such as the muscles of the hindlimb. Thus, our experiments were able to document induction of MuRF1 in muscles not previously recognized as being affected during various atrophic conditions. For example, we observed that dexamethasone induced MuRF1-hiLUCs in the diaphragm and that HLU had particularly strong impacts on the muscles of the tail and lower abdomen (Figure 2C and Figure 5A). Understanding the location, extent and timing of atrophy is an important consideration when assessing the functional performance of animals undergoing muscle loss, and the approach described here readily facilitates the collection of such data.

The central role and universal nature of MuRF1 upregulation during skeletal muscle atrophy [4] made this gene the logical choice for linkage to an optically detectable reporter that could be used with commercially available whole animal imaging platforms. Extensive dissection of tissues from animals undergoing dexamethasone-induced atrophy showed induction patterns that were highly restricted to skeletal muscle with no detectable baseline or induced signal present in heart, lung, small intestine, fat, bone, spleen, or kidney. This profile reinforces the utility of MuRF1-expression as an excellent biomarker for skeletal muscle atrophy. Surprisingly refined details could be ascertained from the whole animal images without the need for further manipulation, for example, the small muscles of the foot were easily identified in hindlimb unloaded animals. Interestingly, work by others has demonstrated that MuRF1 gene expression can also be induced within cardiac muscle during pressure overload in rodent transaortic constriction models [32]. The MuRF1-hiLUCs rats may therefore prove useful in assessing cardiac hypertrophy in response to specific pathological stimuli as well.

Although transgenic reporter systems based on isolated promoter fragments are often sufficient to recapitulate most of the regulatory characteristics of genes, extensive studies in our laboratory indicated that isolated MuRF1 promoter fragments of up to 5 kb in length failed to respond in a manner similar to genomic MuRF1 in numerous cell based assays. This prompted us to pursue a knockin strategy in order to target the 3′ end of the MuRF1 gene with a bicistronic reporter cassette, a configuration meant to harness the MuRF1 regulatory mechanism in its native genomic context without compromising MuRF1 expression itself. This was an important design consideration, since in-depth studies of diseases associated with atrophy are most informative if native atrophic mechanisms remain functionally intact. We carefully analyzed the effects of inserting the luciferase reporter on endogenous MuRF1 expression, and presented multiple lines of evidences that the reporter cassette had a minimal impact on both MuRF1 mRNA and protein level, and the extent of atrophy experienced by the animals subjected to atrophic conditions (Figure 2D, 4, 6 and Figure S2B).

Largely due to technical limitations, most genetically modified rodents reported to date have been generated in mice. Although mouse-based models have many advantages, especially in the logistics of housing and care, there are a multitude of disease models that might benefit from the ability to genetically manipulate rats. For example, diabetes, aging, cardiovascular disease, arthritis, xenobiotic metabolism and numerous neurological disorders may be more closely aligned with the human condition in rats compared to mice [33], [34]. The advent of ZFN-technology has now made it possible to create genetically-modified rats, and the MuRF1-hiLUCs rat is the first published example of a ZFN-mediated knockin of a functional bioluminescent reporter in this species. We envision numerous applications for the MuRF1-hiLUCs reporter rat in studying skeletal muscle atrophy, both in basic research and in drug discovery workflows. Since MuRF1 is considered to be a general marker for skeletal muscle atrophy, our transgenic reporter animals provide an attractive tool to investigate a variety of diseases and/or conditions with atrophy components such as aging, cancer, chronic heart failure, chronic kidney disease, diabetes, or ventilator-induced diaphragmatic dysfunction.

## Supporting Information

Figure S1 Donor plasmid configuration, PCR verification of insertion site and junction sequencing of knockin reporter. (**A**) Strategy for creating the MuRF1-hiLUCs knockin rat utilizing a donor plasmid with homology arms. An HCV- IRES-Luciferase-2A-SEAP reporter was inserted into the last intron of the endogenous MuRF1 gene via ZFN targeting. Primer sites for PCR detection of site-specific insertion or random integration depicted as numbered arrows. (**B**) PCR and sequence verification of the targeted insertion of the MuRF1-hiLUCs donor plasmid. PCR primers used to verify targeted insertion and rule out random integration were shown in (**A**).
(TIF)

Figure S2 Intact MuRF1 genomic sequence and unaltered protein expression in MuRF1-hiLUCs reporter rat. (**A**) Genomic DNA sequence of the MuRF1-hiLUCs allele spanning from intron 8–9 of the MuRF1 gene to the end of the luciferase ORF. Purple: 3′ end of Intron 8–9 of the MuRF1 gene; Red: Exon 9 of the MuRF1 gene; Gray: Linker between MuRF1 and IRES; Blue: HCV-IRES; Green: Luciferase ORF. (**B**) Western blot analysis of the MuRF1 protein expression in heterozygous reporter rats compared to *wild-type* rats. No difference in the expression level or banding pattern was observed.
(TIF)

Figure S3 Induction of the MuRF1-hiLUCs mRNA in the reporter rat after 7 days of dexamethasone treatment. Reporter rats were given 1.5 mg/kg/day of dexamethasone in drinking water. Gastrocnemius muscles were harvested on day 7 and used for RNA preparation. Quantitative RT-PCR shows similar induction levels of MuRF1-hiLUCs and luciferase mRNAs in the heterozygous reporter rats following dexamethasone treatment. MuRF1-hiLUCs mRNA was amplified using an allele specific primer set: the forward primer sequence lies in the exon 8-9 boundary of the MuRF1 gene and the reverse primer lies in the linker sequence between the MuRF1 gene and the IRES sequence (Supplemental Figure 2A). Data are presented as mean (symbols) + SEM (lines). Statistics were performed using one-way ANOVA with Tukey's multiple comparisons test. ****P<0.0001.
(TIF)

Figure S4 Induction of MuRF1-hiLUCs reporter activity and endogenous MuRF1 expression following dexamethasone treatment. (**A**) Dorsal images of the same rat shown in Figure 2a. (**B**) Quantitation of the fold induction of luminescent intensity of the MuRF1-hiLUCs reporter during the time course of the dexamethasone (n = 10 for day 1–5, n = 9 for day 7) or saline (n = 8) treatment. Five dorsal ROIs were used for the quantitation. Data are presented as mean (symbols) + SEM (lines). Statistics were performed using 2way ANOVA with Dunnett's multiple comparisons test. *P<0.05, ***P<0.001, ****P<0.0001. (**C**) qRT-PCR shows induction of MuRF1 and luciferase mRNA in the gastrocnemius muscles after 7 days of dexamethasone treatment. Data are presented as mean (symbols) + SEM (lines). Statistics were performed using one-way ANOVA with Tukey's multiple comparisons test. ****P<0.0001.
(TIF)

Figure S5 Representative images of a denervated and a sham-operated MuRF1-hiLUCs rat throughout the time course of a 14-day hindlimb denervation study. (**A**) Left lateral images of the same two rats show in Figure 3A. (**B**) Right lateral images of the same two rats show in Figure 3A.
(TIF)

Figure S6 Extent of muscle atrophy after 14-day denervation in MuRF1-hiLUCs reporter rats compared to *wild-type* rats. Left panel, the same data from Figure 4A was re-analyzed and muscle weights from the denervated left leg were normalized to body weights instead of the muscle weights from the contralateral right leg. Right panel, muscle weights from the contralateral right leg were compared between sham-operated, denervated reporter and denervated *wild-type* rats, no significant differences were observed. Data are presented as means (symbols) + SEM (lines). Statistics were performed using ordinary one-way ANOVA with Tukey's multiple comparisons test. **P<0.01, ****P<0.0001.
(TIF)

Figure S7 MuRF1-hiLUCs reporter induction in loaded control rats during a 14-day HLU study. Loaded control rats (n = 6) were taped around their tails and singly housed in the same cage environment as HLU rats for 14 days. Imaging data from control rats (n = 6) were obtained and quantified the same way as HLU rats.
(TIF)

Table S1 Induction of MuRF1-hiLUCs in different ROIs during the time course of a 14-day HLU study. Fold inductions of the MuRF-hiLUCs reporter in HLU rats (n = 8) were quantified as described in Figure 5. ROIs in lower body to upper body are arranged in descending order in the table. All three views showed significant induction of MuRF1-hiLUCs in the hindquarter of the body that was sustained throughout the time course of the study. In contrast, MuRF1-hiLUCs induction in the upper body ROIs were significant on day 1–5 but returned to baseline on day 7–14 of the time course. ****P<0.0001, ***P<0.001, **P<0.01, *P<0.05.
(TIF)
