# Supplementary figures and images for: Noninvasive Imaging of In Vivo MuRF1 Expression during Muscle Atrophy
Source: PLoS One. 2014 Apr 7;9(4):e94032. doi: 10.1371/journal.pone.0094032 (PMC3977994; doi:10.1371/journal.pone.0094032)

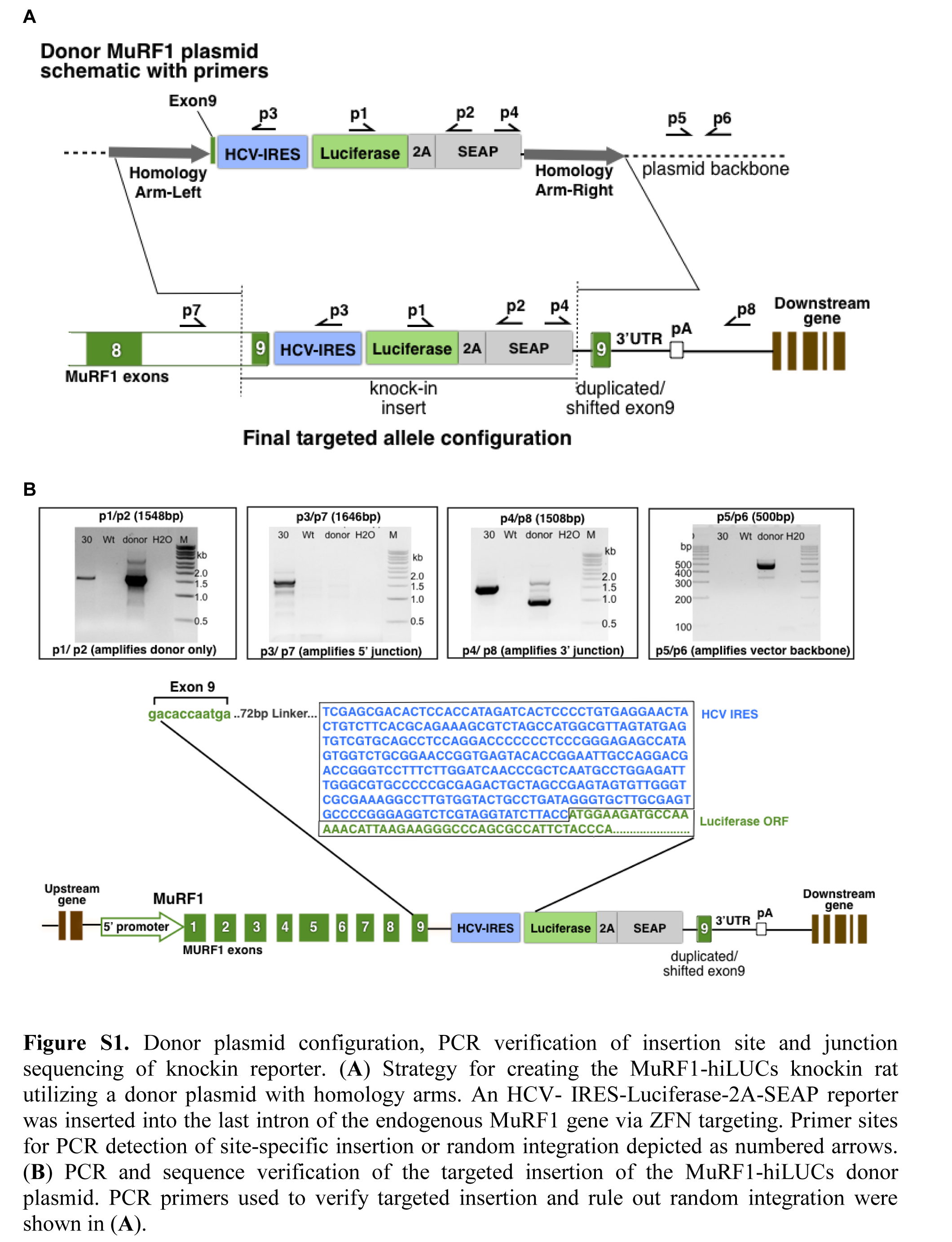

Supplement: Figure S1 — Donor plasmid configuration, PCR verification of insertion site and junction sequencing of knockin reporter. (A) Strategy for creating the MuRF1-hiLUCs knockin rat utilizing a donor plasmid with homology arms. An HCV- IRES-Luciferase-2A-SEAP reporter was inserted into the last intron of the endogenous MuRF1 gene via ZFN targeting. Primer sites for PCR detection of site-specific insertion or random integration depicted as numbered arrows. (B) PCR and sequence verification of the targeted insertion of the MuRF1-hiLUCs donor plasmid. PCR primers used to verify targeted insertion and rule out random integration were shown in (A). (TIF) [file pone.0094032.s001.tif]

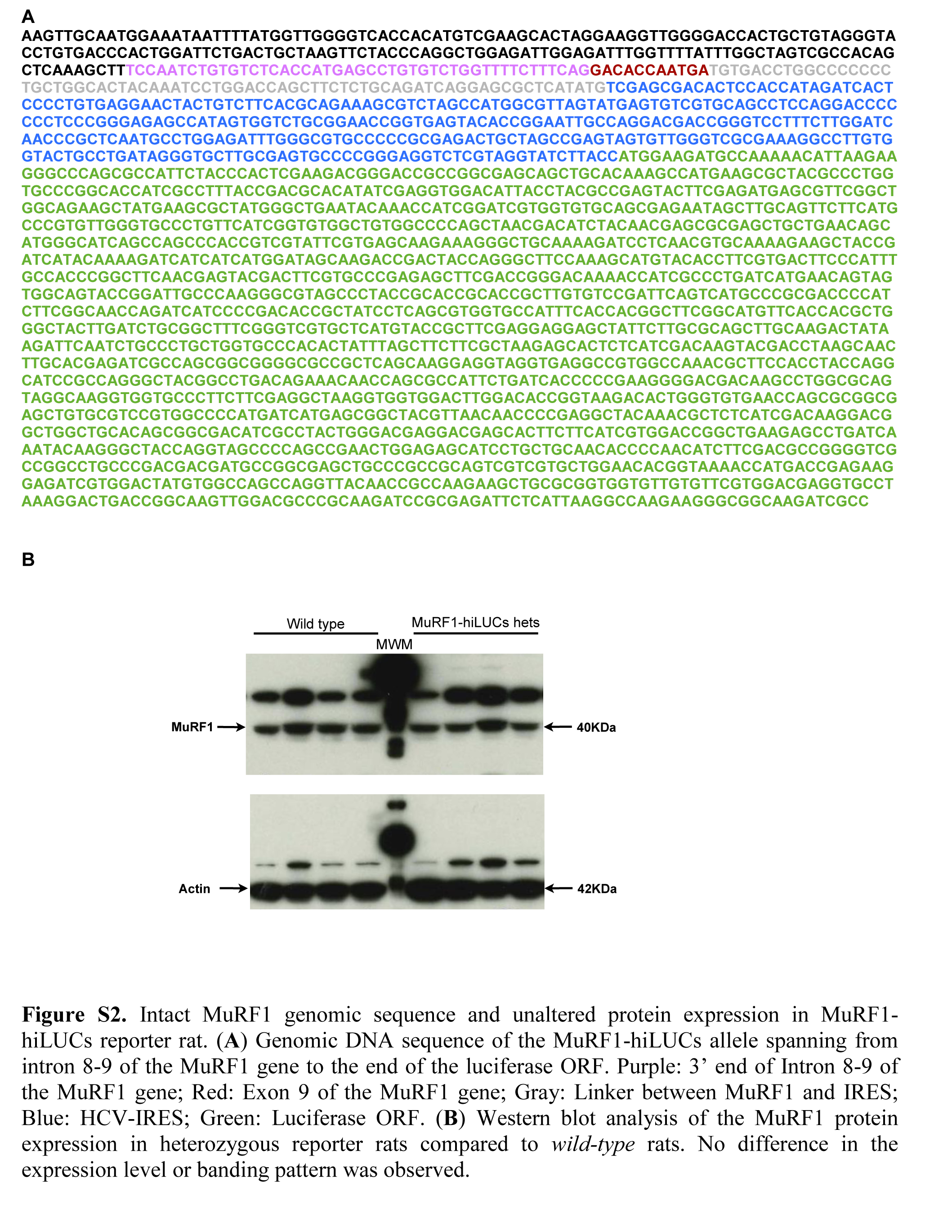

Supplement: Figure S2 — Intact MuRF1 genomic sequence and unaltered protein expression in MuRF1-hiLUCs reporter rat. (A) Genomic DNA sequence of the MuRF1-hiLUCs allele spanning from intron 8–9 of the MuRF1 gene to the end of the luciferase ORF. Purple: 3′ end of Intron 8–9 of the MuRF1 gene; Red: Exon 9 of the MuRF1 gene; Gray: Linker between MuRF1 and IRES; Blue: HCV-IRES; Green: Luciferase ORF. (B) Western blot analysis of the MuRF1 protein expression in heterozygous reporter rats compared to wild-type rats. No difference in the expression level or banding pattern was observed. (TIF) [file pone.0094032.s002.tif]

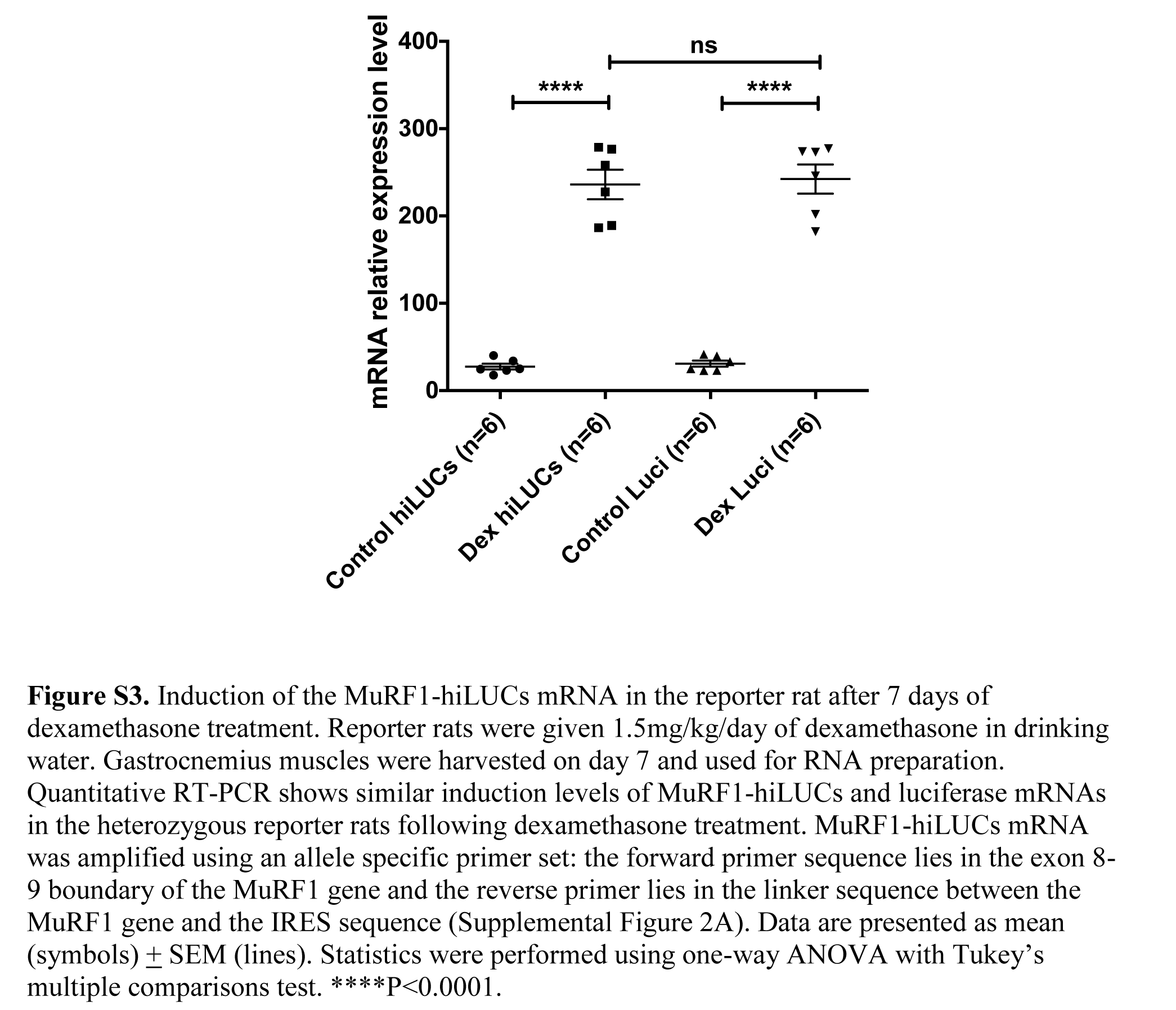

Supplement: Figure S3 — Induction of the MuRF1-hiLUCs mRNA in the reporter rat after 7 days of dexamethasone treatment. Reporter rats were given 1.5 mg/kg/day of dexamethasone in drinking water. Gastrocnemius muscles were harvested on day 7 and used for RNA preparation. Quantitative RT-PCR shows similar induction levels of MuRF1-hiLUCs and luciferase mRNAs in the heterozygous reporter rats following dexamethasone treatment. MuRF1-hiLUCs mRNA was amplified using an allele specific primer set: the forward primer sequence lies in the exon 8-9 boundary of the MuRF1 gene and the reverse primer lies in the linker sequence between the MuRF1 gene and the IRES sequence (Supplemental Figure 2A). Data are presented as mean (symbols) + SEM (lines). Statistics were performed using one-way ANOVA with Tukey's multiple comparisons test. ****P<0.0001. (TIF) [file pone.0094032.s003.tif]

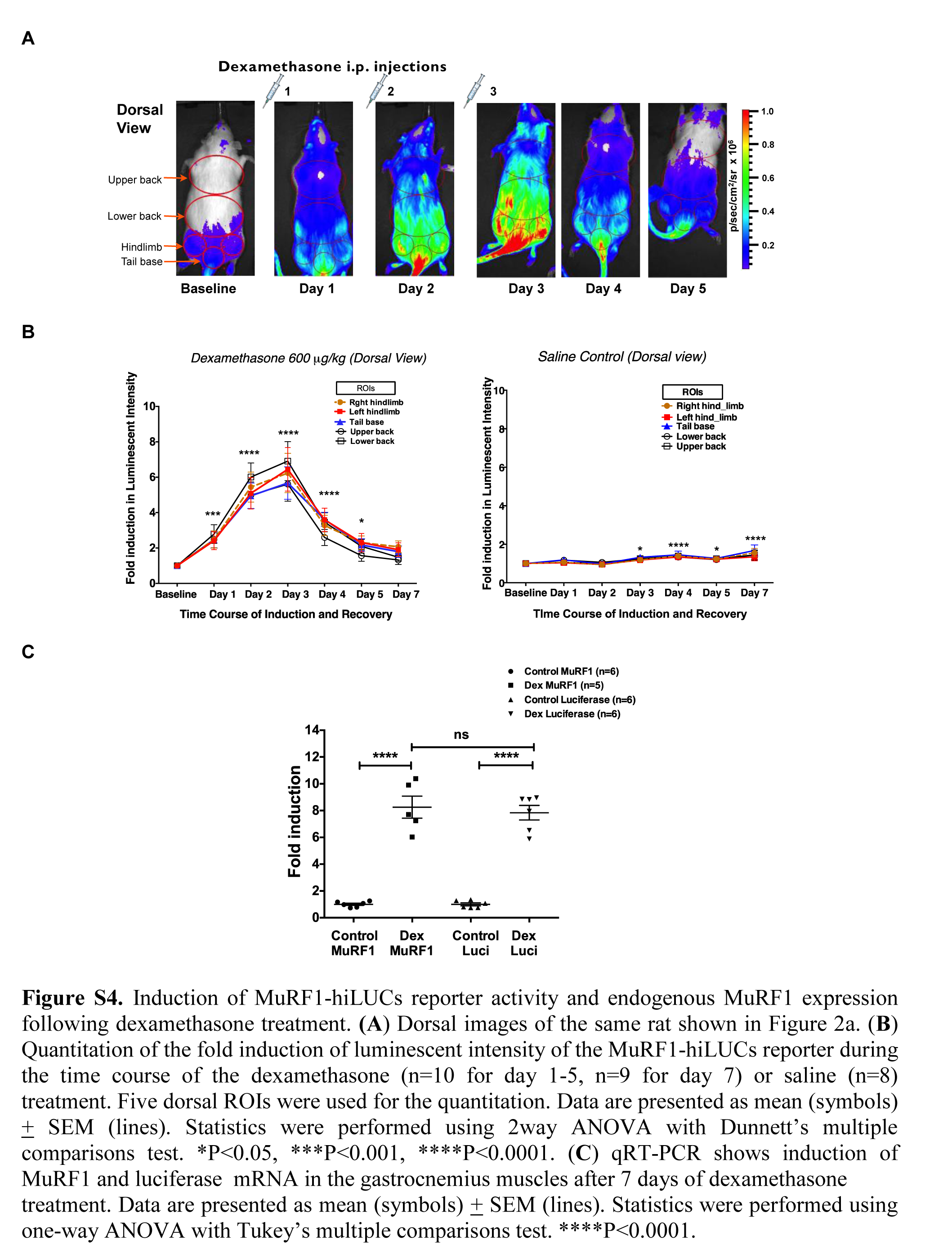

Supplement: Figure S4 — Induction of MuRF1-hiLUCs reporter activity and endogenous MuRF1 expression following dexamethasone treatment. (A) Dorsal images of the same rat shown in Figure 2a. (B) Quantitation of the fold induction of luminescent intensity of the MuRF1-hiLUCs reporter during the time course of the dexamethasone (n = 10 for day 1–5, n = 9 for day 7) or saline (n = 8) treatment. Five dorsal ROIs were used for the quantitation. Data are presented as mean (symbols) + SEM (lines). Statistics were performed using 2way ANOVA with Dunnett's multiple comparisons test. *P<0.05, ***P<0.001, ****P<0.0001. (C) qRT-PCR shows induction of MuRF1 and luciferase mRNA in the gastrocnemius muscles after 7 days of dexamethasone treatment. Data are presented as mean (symbols) + SEM (lines). Statistics were performed using one-way ANOVA with Tukey's multiple comparisons test. ****P<0.0001. (TIF) [file pone.0094032.s004.tif]

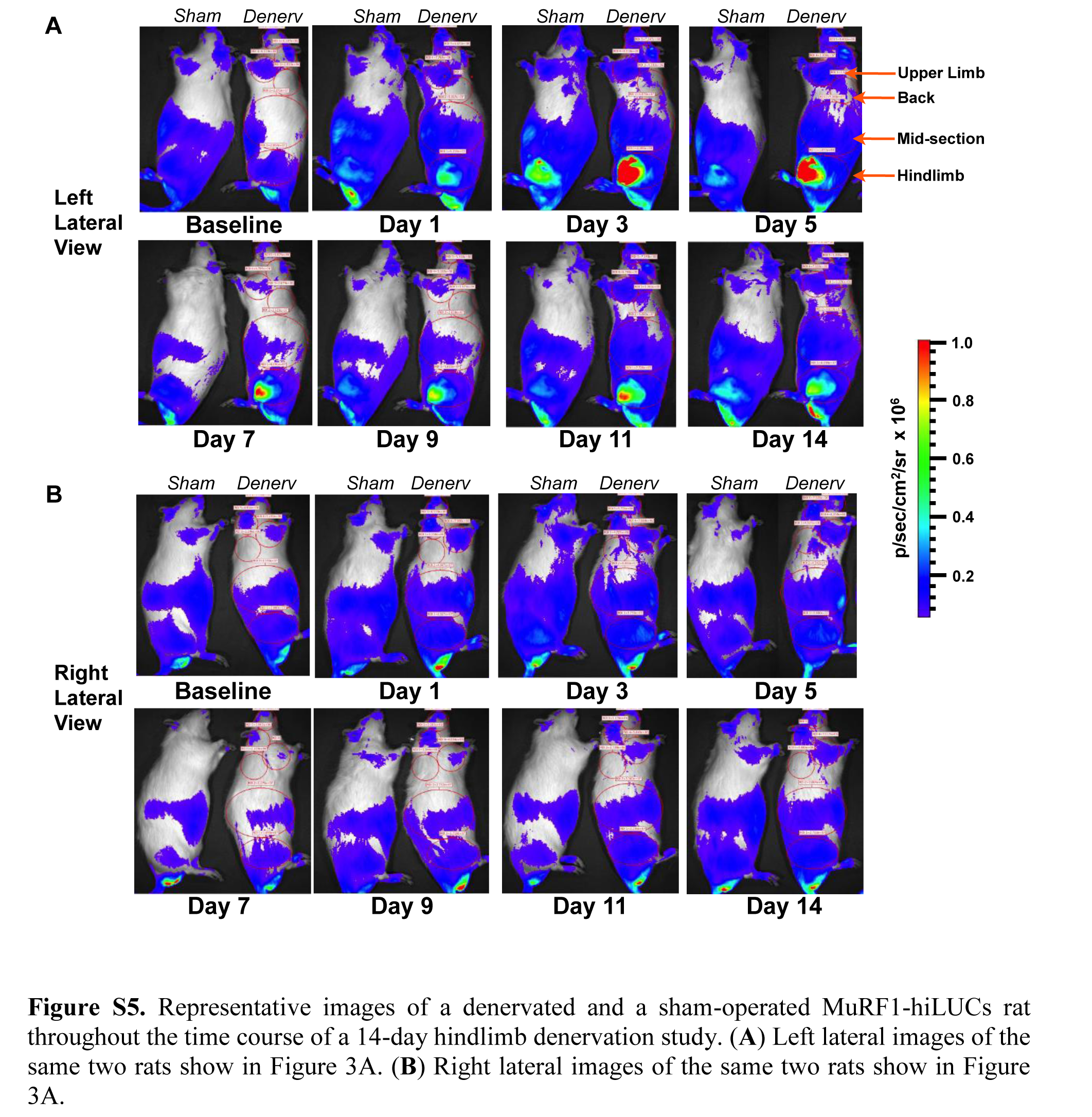

Supplement: Figure S5 — Representative images of a denervated and a sham-operated MuRF1-hiLUCs rat throughout the time course of a 14-day hindlimb denervation study. (A) Left lateral images of the same two rats show in Figure 3A. (B) Right lateral images of the same two rats show in Figure 3A. (TIF) [file pone.0094032.s005.tif]

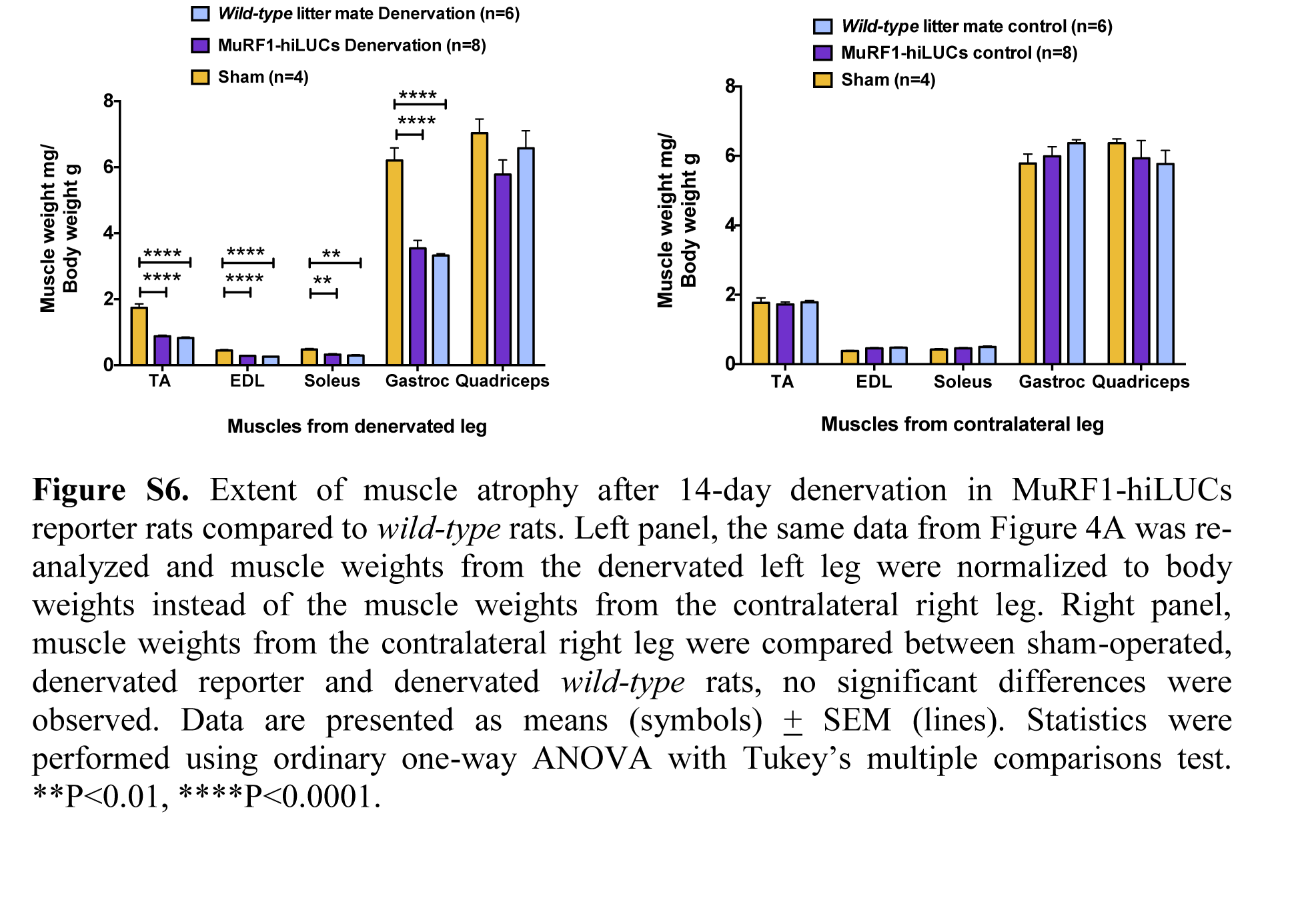

Supplement: Figure S6 — Extent of muscle atrophy after 14-day denervation in MuRF1-hiLUCs reporter rats compared to wild-type rats. Left panel, the same data from Figure 4A was re-analyzed and muscle weights from the denervated left leg were normalized to body weights instead of the muscle weights from the contralateral right leg. Right panel, muscle weights from the contralateral right leg were compared between sham-operated, denervated reporter and denervated wild-type rats, no significant differences were observed. Data are presented as means (symbols) + SEM (lines). Statistics were performed using ordinary one-way ANOVA with Tukey's multiple comparisons test. **P<0.01, ****P<0.0001. (TIF) [file pone.0094032.s006.tif]

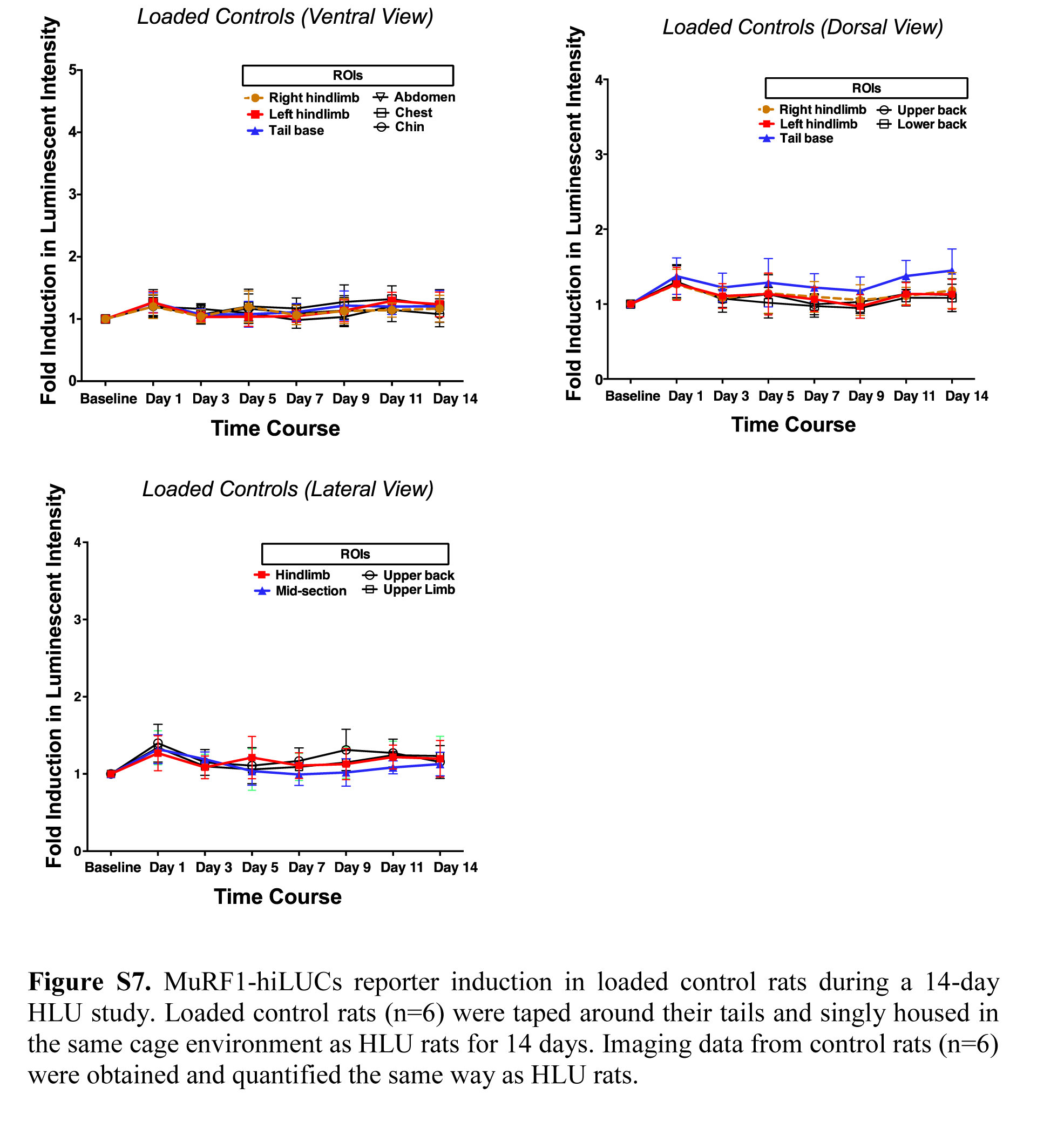

Supplement: Figure S7 — MuRF1-hiLUCs reporter induction in loaded control rats during a 14-day HLU study. Loaded control rats (n = 6) were taped around their tails and singly housed in the same cage environment as HLU rats for 14 days. Imaging data from control rats (n = 6) were obtained and quantified the same way as HLU rats. (TIF) [file pone.0094032.s007.tif]

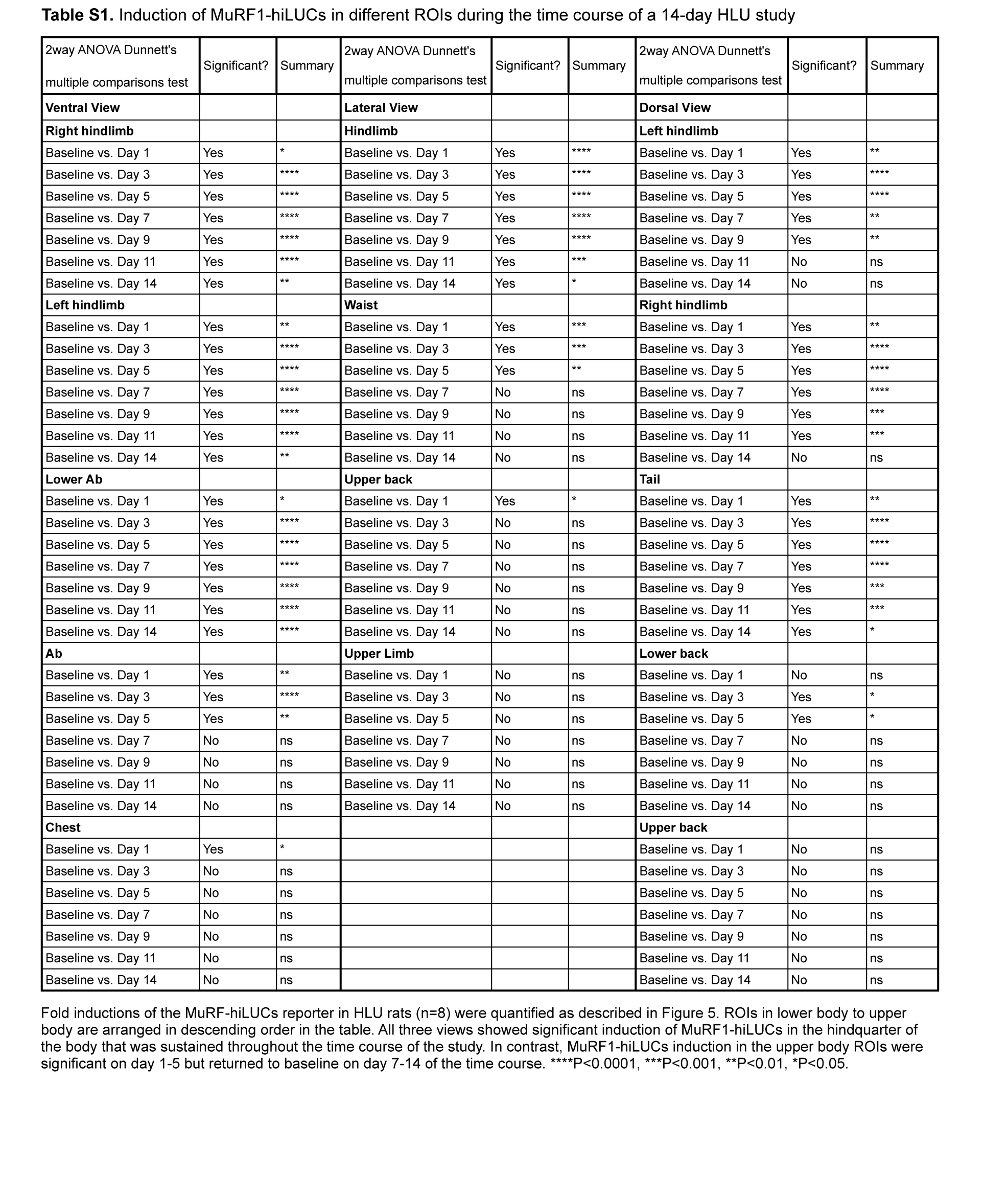

Supplement: Table S1 — Induction of MuRF1-hiLUCs in different ROIs during the time course of a 14-day HLU study. Fold inductions of the MuRF-hiLUCs reporter in HLU rats (n = 8) were quantified as described in Figure 5. ROIs in lower body to upper body are arranged in descending order in the table. All three views showed significant induction of MuRF1-hiLUCs in the hindquarter of the body that was sustained throughout the time course of the study. In contrast, MuRF1-hiLUCs induction in the upper body ROIs were significant on day 1–5 but returned to baseline on day 7–14 of the time course. ****P<0.0001, ***P<0.001, **P<0.01, *P<0.05. (TIF) [file pone.0094032.s008.tif]
